# Towards Highly Performing and Stable PtNi Catalysts in Polymer Electrolyte Fuel Cells for Automotive Application

**DOI:** 10.3390/ma10030317

**Published:** 2017-03-21

**Authors:** Sabrina C. Zignani, Vincenzo Baglio, David Sebastián, Ada Saccà, Irene Gatto, Antonino S. Aricò

**Affiliations:** CNR-Istituto di Tecnologie Avanzate per l’Energia “Nicola Giordano”, Via Salita S. Lucia sopra Contesse 5, 98126 Messina, Italy; zignani@itae.cnr.it (S.C.Z.); sebastian@itae.cnr.it (D.S.); sacca@itae.cnr.it (A.S.); gatto@itae.cnr.it (I.G.); arico@itae.cnr.it (A.S.A.)

**Keywords:** PtNi/C catalyst, oxygen reduction reaction, polymer electrolyte fuel cell, accelerated degradation test

## Abstract

In order to help the introduction on the automotive market of polymer electrolyte fuel cells (PEFCs), it is mandatory to develop highly performing and stable catalysts. The main objective of this work is to investigate PtNi/C catalysts in a PEFC under low relative humidity and pressure conditions, more representative of automotive applications. Carbon supported PtNi nanoparticles were prepared by reduction of metal precursors with formic acid and successive thermal and leaching treatments. The effect of the chemical composition, structure and surface characteristics of the synthesized samples on their electrochemical behavior was investigated. The catalyst characterized by a larger Pt content (Pt_3_Ni_2_/C) presented the highest catalytic activity (lower potential losses in the activation region) among the synthesized bimetallic PtNi catalysts and the commercial Pt/C, used as the reference material, after testing at high temperature (95 °C) and low humidification (50%) conditions for automotive applications, showing a cell potential (ohmic drop-free) of 0.82 V at 500 mA·cm^−2^. In order to assess the electro-catalysts stability, accelerated degradation tests were carried out by cycling the cell potential between 0.6 V and 1.2 V. By comparing the electrochemical and physico-chemical parameters at the beginning of life (BoL) and end of life (EoL), it was demonstrated that the Pt_1_Ni_1_/C catalyst was the most stable among the catalyst series, with only a 2% loss of voltage at 200 mA·cm^−2^ and 12.5% at 950 mA·cm^−2^. However, further improvements are needed to produce durable catalysts.

## 1. Introduction

Fuel cell technology is very close to the market application; however, a further optimization and a decrease of costs are still necessary [1,2]. Furthermore, for an easy thermal and water management, the automotive fuel cell market requires an increase of the operating temperature (in the range 90–130 °C) and operation with a relative humidity (R.H.) less than 50% [3]. In order to increase the performance and reduce the Pt content in the electrodes, several approaches have been pursued, such as the use of binary and ternary Pt-alloys, e.g., PtCo, PtNi, PtCoCr, PtCoMn [4,5,6,7,8,9,10,11], and, more recently, in order to decrease the cost, platinum group metal-free (PGM-free) catalysts [12,13,14].

Several works reported an enhancement of the oxygen reduction reaction (ORR) activity by factors of 1.5 to 3 for Pt-alloys in comparison to pure Pt, due to electronic and structural effects. One of the most promising and studied formulations is Pt-Ni [15,16,17,18,19,20,21,22], with mass activities exceeding those of carbon supported high surface area Pt and Pt-alloy catalysts [23]. Stamenkovic et al. [24] reported that the (111) surface of Pt_3_Ni exhibits a 10-fold higher ORR activity than Pt (111) and a 90-fold higher ORR activity than commercial Pt/C catalysts. Although several mechanisms have been proposed to explain the high ORR activity of Pt–Ni structures [25,26], the best composition is still not clear, in particular under practical fuel cell conditions. Furthermore, these catalysts show insufficient durability, and suffer from a loss of structural integrity by metal segregation and de-alloying [16]. These limitations may be addressed by developing well-defined catalyst compositions and structures using proper preparation procedures. Some preparation procedures can effectively produce stable catalysts with an enrichment of Pt in the outermost layers of alloyed bimetallic catalysts [7,27,28]. These regard an induced surface segregation of Pt by high-temperature annealing and a removal of the less noble transition metal from the alloy surface by pre-leaching in an appropriate acid. This approach also produces better electrochemical activity, as reported in the literature [7,27,28]. Accordingly, in this work, we have prepared various PtNi/C catalysts, characterized by different atomic compositions, by using the formic acid reduction method and successive annealing treatment at 900 °C. We have used this preparation procedure because it is simpler, allowing the simultaneous reduction of Pt and Ni in a single step, and less time-consuming than other preparation procedures reported in the literature (i.e., sulphite–complex route) [3], in which the preparation of PtNi/C catalyst generally comprises at least two processes concerning first Pt/C synthesis and then Ni addition to Pt nanoparticles, with one or more thermal treatment and acid leaching steps. A leaching procedure in 0.5 M HClO_4_ was carried out for the synthesized PtNi catalysts after the thermal treatment to remove the non-alloyed Ni atoms from the surface. To shed a light on the better atomic composition of Pt-Ni electrocatalysts, the samples were electrochemically investigated in a polymer electrolyte fuel cell (PEFC) in terms of performance and stability to accelerated degradation tests. Despite the potential application of PtNi/C catalysts in PEFC, few works in the literature report on the performance evaluation under realistic conditions [8,29,30,31]. The main objective of this work is thus to investigate PtNi/C catalysts in a PEFC under low relative humidity and pressure conditions, more representative of automotive applications.

## 2. Results and Discussion

### 2.1. Physico-Chemical Characterization

**Figure 1:**
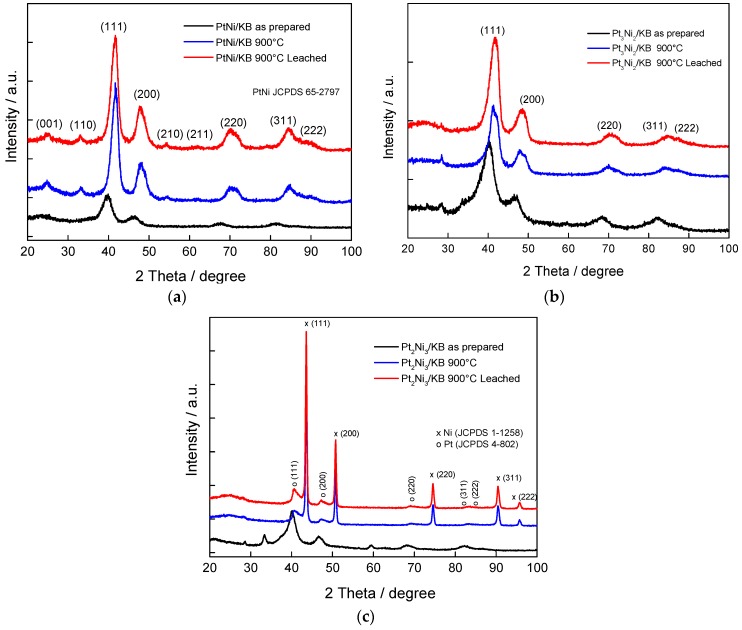
XRD patterns of carbon supported (**a**) Pt_1_Ni_1_; (**b**) Pt_3_Ni_2_; and (**c**) Pt_2_Ni_3_ catalysts (as-prepared, treated at 900 °C and leached).

Figure 1 shows the X-ray diffraction (XRD) patterns of carbon supported Pt_1_Ni_1_/KB, Pt_3_Ni_2_/KB and Pt_2_Ni_3_/KB as prepared, treated at 900 °C and leached after the thermal treatment. XRD patterns of the Pt_1_Ni_1_/KB, reported in Figure 1a show a disordered cubic structure (fcc) for the as-prepared catalyst and a single ordered primitive cubic (L1_2_) phase for the alloy treated at 900 °C. The occurrence of the primitive cubic structure in the sample treated at high temperature is evident from the presence of the superlattice reflections, i.e., (001), (110) and (210) and from a better matching with the Joint Committee on Powder Diffraction Standards (JCPDS) card (65-2797) than the JCPDS card (04-0802) related to Pt. The as-prepared catalyst does not show any significant separation between Ni and Pt probably due to the low level of crystallinity, and after the high temperature treatment (900 °C), no separation of metallic Ni phase was observed. A shift towards higher Bragg angles is observed, indicating the formation of the solid solution between Pt and Ni, with a 35 at % of Ni in the alloy (Table 1). As reported in Table 1, the crystallite size after the thermal treatment is about 2.7 nm.

The catalyst prepared with an excess of Pt (Pt_3_Ni_2_/KB), whose XRD patterns are reported in Figure 1b, shows small crystallites, about 2.3 nm, but also the presence of Ni hydroxide species for the as-prepared catalyst (see the shoulder at about 35° 2θ) that evolve with the formation of a separate metallic Ni phase at 900 °C (not clearly visible, since the Ni peaks are very close to Pt reflections). Thereafter, the effect of leaching was investigated. This post-treatment promoted the dissolution of unalloyed Ni, as proven by the XRD analysis. Nonetheless, a suitable alloying (close to the bulk composition, see Table 1) was achieved as proven by the shift of fcc reflections. 

In the case of the catalyst containing an excess of Ni (Pt_2_Ni_3_/C), whose XRD patterns are reported in Figure 1c, the presence of sharp peaks indicating large crystallites of metallic Ni after the thermal treatment (JCPDS card 1-1258) is evident. For the as-prepared catalyst, the presence of Ni hydroxides is clearly evident. After the thermal treatment at 900 °C, the formation of two separate metallic phases occurs, one rich in Ni and the other in Pt. After the leaching post-treatment, only a slightly modification is achieved and the two separate phases are still present. However, a certain degree of alloying is obtained (26% Ni in the alloy). The crystallite size for the Pt phase is larger than 6 nm, as reported in Table 1.

**Figure 2:**
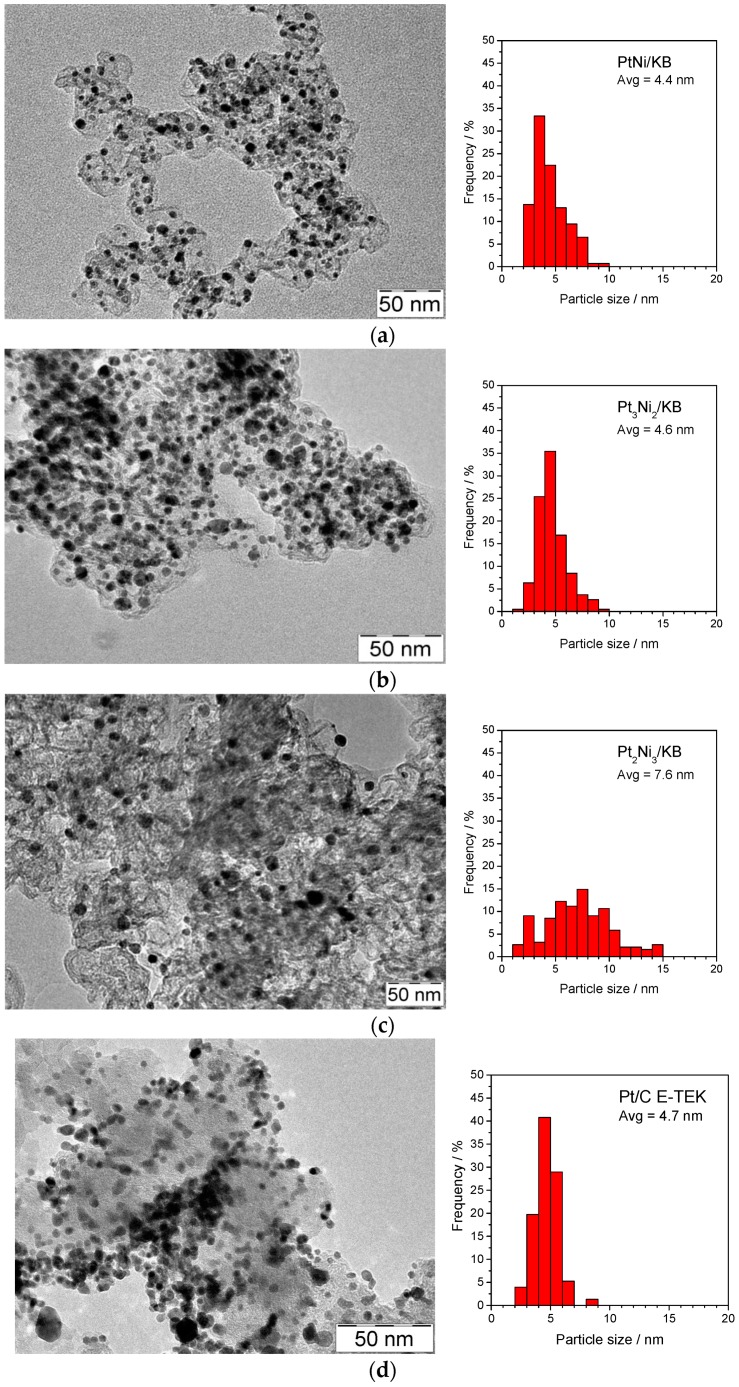
Transmission electron micrographs (TEM) analysis of carbon supported (**a**) Pt_1_Ni_1_; (**b**) Pt_3_Ni_2_; (**c**) Pt_2_Ni_3_ catalysts after the thermal and leaching treatments and (**d**) Pt/C E-TEK catalyst.

A morphological analysis of the dispersion of the metallic particles on carbon (Figure 2) shows an increase of the particle size for the sample with a larger Ni content (average Pt particle size of 7.6 nm). Particle sizes around 4.5 nm are observed for the Pt_1_Ni_1_ and Pt_3_Ni_2_ treated at 900 °C. On the other hand, the commercial Pt/C catalyst (E-TEK) exhibits a narrow particle size distribution with average size of 4.7 nm.

**Figure 3:**
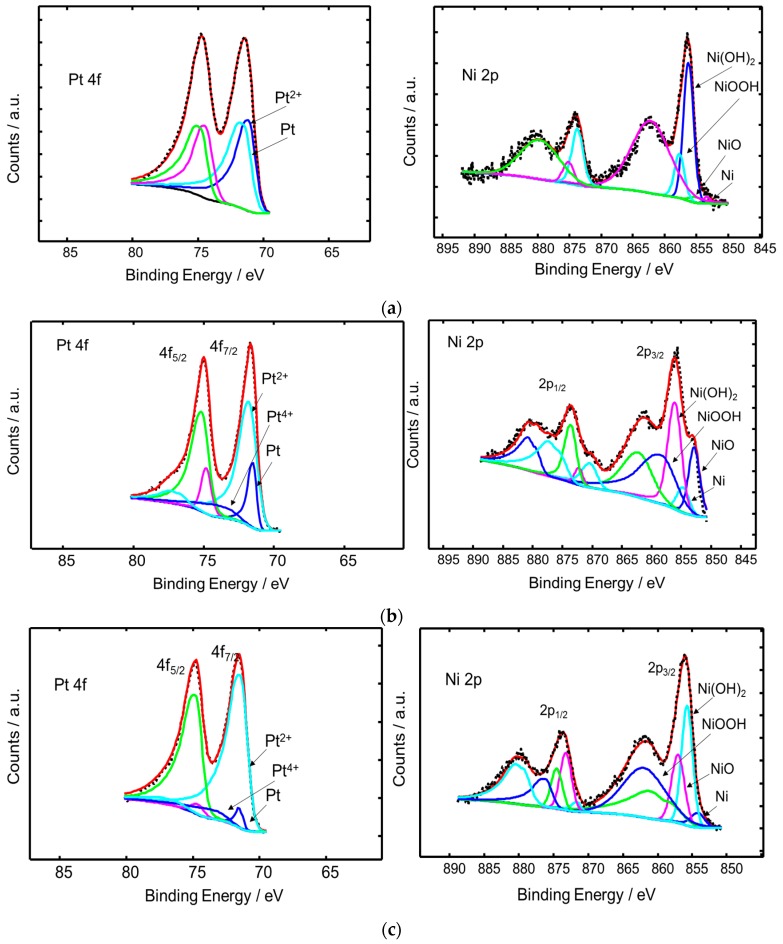
XPS spectra of Pt 4f and Ni 2p signals for carbon supported (**a**) Pt_1_Ni_1_; (**b**) Pt_3_Ni_2_; and (**c**) Pt_2_Ni_3_ catalysts after the thermal and leaching treatments.

A surface characterization of the Pt-Ni samples was carried out by X-ray photoelectron spectroscopy (XPS). There was no significant change in the surface composition of the samples compared to the Pt:Ni ratio of their bulk compositions as evaluated by the energy dispersive X-ray analysis (EDX) analysis (not shown). Figure 3a shows the Pt 4f and Ni 2p spectra of the Pt_1_Ni_1_/C sample as prepared. In the case of Pt, the analysis of the photoelectron spectra indicates similar occurrence of metallic and oxidized Pt (2+) on the surface. Regarding Ni 2p, the deconvolution of the main bands shows a prevalence of hydroxide and oxide species; whereas, a very low amount of metallic Ni is present in the surface. The presence of such hydroxide and oxide species in the outermost layers may be due to the oxidation of surface Ni species.

The XPS analysis of the sample containing an excess of Pt (Pt_3_Ni_2_/C) is reported in Figure 3b. Based on the quantitative determination reported in Table 2, it is possible to state that this sample shows a more oxidised Pt on the surface than the Pt_1_Ni_1_. Furthermore, this sample shows that Ni is, also in this case, largely present as hydroxide and oxide species on the surface with a small amount of zero-valent Ni.

The XPS analysis of the sample containing an excess of Ni (Pt_2_Ni_3_/C) shows that Pt is essentially occurring on the surface as Pt^2+^, whereas the Ni occurs as hydroxide and oxide species (Figure 3c). A complete dataset of the surface composition and surface oxidation states of the investigated samples is reported in Table 2.

### 2.2. Electrochemical Characterization

**Figure 4:**
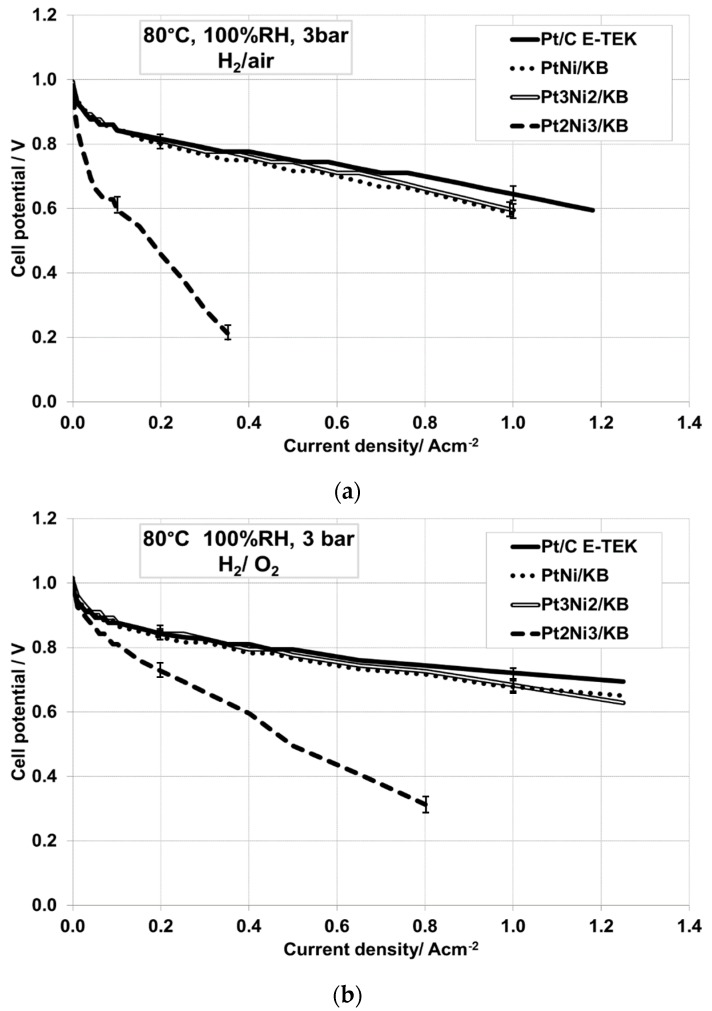
Polarization curves in (**a**) H_2_/air and (**b**) H_2_/O_2_ among the MEAs equipped with the different cathodic PtNi catalysts and the benchmark Pt/C catalyst at 80 °C, 100% relative humidity (RH), 3 bar_abs_.

The electrochemical performance of the PtNi/C cathodic catalysts prepared by the formic acid method and successive thermal and leaching treatments was evaluated in a single cell based on a thin Nafion NR212 membrane. This was selected in order to increase the operating temperature of the PEFC device, reducing simultaneously the relative humidity (R.H.) and pressure to simulate the automotive conditions. However, initially, the cells were conditioned and investigated at 80 °C and full humidification (100% R.H., 3 bar_abs_) as reference conditions. The polarization curves under the latter conditions and in the presence of air as the oxidant are reported in Figure 4a. They show a similar performance for Pt_1_Ni_1_/C, Pt_3_Ni_2_/C and the benchmark Pt/C catalyst, in particular, in the low current density (activation) region; whereas, at high current density, the cell equipped with the benchmark catalyst appears slightly better performing than the other catalysts. On the other hand, the behavior of the catalyst rich in Ni and with the presence of large metallic Ni particles was not showing a performance level than the other cathode catalysts investigated (Figure 4). This was due to the large particle size and the presence of two separate phases (PtNi and metallic Ni). The presence of the Ni phase can be also responsible of the high cell resistance recorded for the cell based on this catalyst, as can be observed in Table 3.

**Figure 5:**
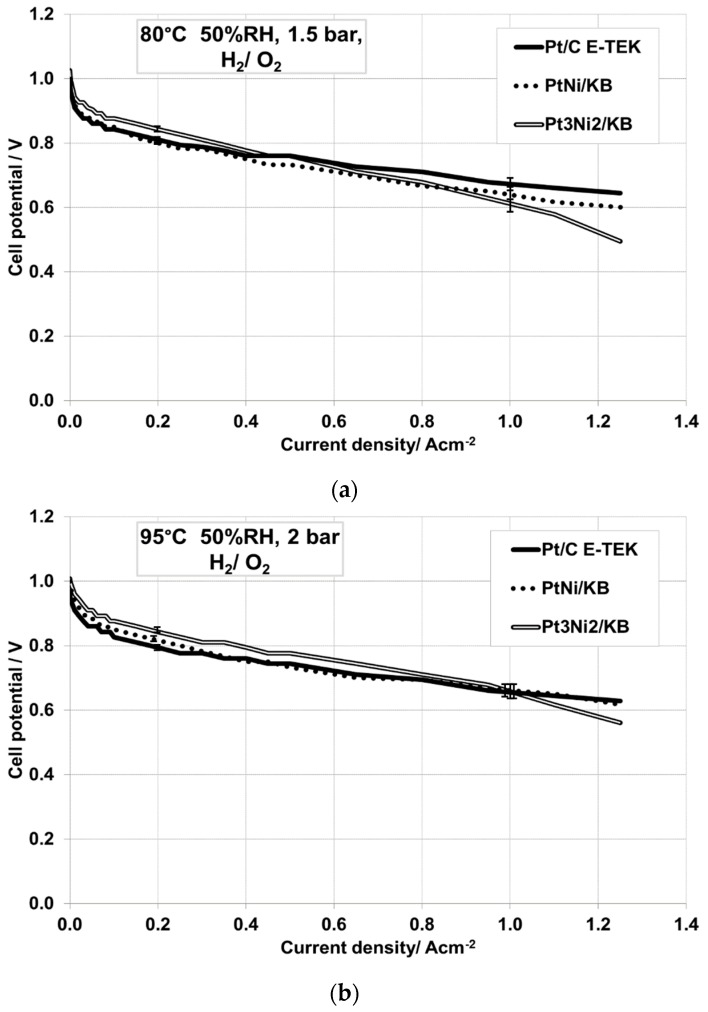
Polarization curves among the MEAs equipped with the different cathodic PtNi catalysts and the benchmark Pt/C catalyst at different operative conditions: (**a**) 80 °C, 50% R.H., 1.5 bar_abs_, H_2_/O_2_; and (**b**) 95 °C, 50% R.H., 2 bar_abs_, H_2_/O_2_.

In order to exacerbate the differences among the curves, oxygen was fed at the cathode and the polarization curves (Figure 4b) were also recorded under these conditions (80 °C, 100% R.H., 3 bar_abs_). The observed trend is exactly the same of that recorded by feeding air at the cathode (see Figure 4a,b). Since the cell based on the cathode catalyst that was rich in Ni showed a poor performance, this cell was not investigated further; however, the membrane electrode assemblies (MEAs) based on Pt_1_Ni_1_/C and Pt_3_Ni_2_/C were tested at 80 °C under low humidification (50% R.H., 1.5 bar_abs_) conditions (Figure 5a) and compared to the one equipped with the commercial Pt/C catalyst.

At low RH, the MEA based on the Pt-rich catalyst (Pt_3_Ni_2_/C) showed a lower voltage loss in the activation region, compared to the equimolar bimetallic catalyst and benchmark Pt/C. The slightly better behavior of this catalyst could be ascribed to a proper composition or to a lower crystallite size compared to the equimolar PtNi sample. On one hand, the Pt crystallite size of Pt_3_Ni_2_/KB (2.3 nm, Table 1) is slightly lower than PtNi/KB (2.7 nm). A smaller particle size means larger electrochemical surface area and more available active sites for the oxygen reduction. However, since negligible differences were observed between PtNi catalysts under full humidification, the different surface characteristics could also play a role in enhancing the catalytic activity of the catalyst. The most relevant characteristic of Pt_3_Ni_2_/KB is its larger amount of metallic Ni (19.7%) as revealed by XPS, which could contribute to oxygen adsorption on the Pt surface (by geometric and electronic effects). The polarization curves recorded at 95 °C under low R.H. conditions (Figure 5b) confirm a better catalytic activity of the Pt-rich bimetallic catalyst compared to the other ones. In this case, the PtNi (1:1) also shows a higher catalytic activity than the Pt/C, indicating the beneficial effect of Ni in enhancing the kinetics of the oxygen reduction reaction, in particular under low humidification. The proton availability related to the water content has, of course, an effect on the kinetics. A significant reduction of relative humidity caused a decrease of proton availability and a consequent increase of the activation barrier for the ORR. This aspect could be associated to both membrane and ionomer dry-out, resulting in a lower availability of protons at the catalyst-ionomer electrolyte interface. The presence of Ni, in its oxidized forms, could help to reduce these constraints.

**Figure 6:**
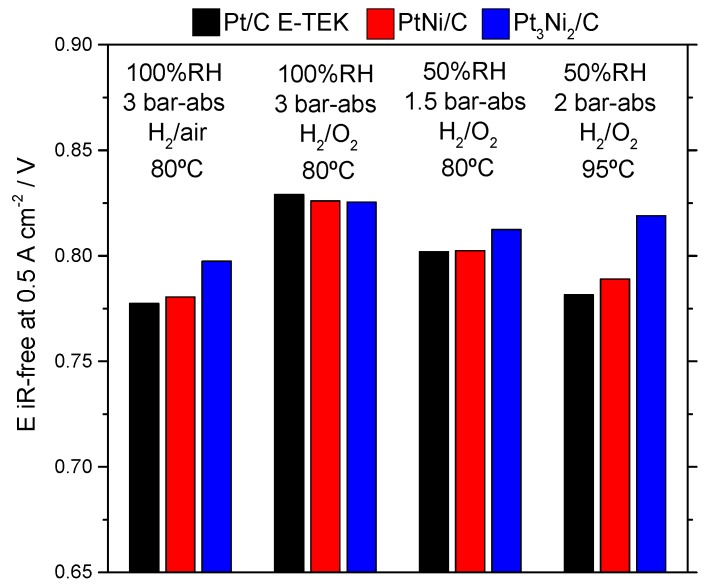
Cell potential (iR-free) at 0.5 A·cm^−2^ for the MEAs equipped with PtNi/C (red), Pt_3_Ni_2_/C (blue) and Pt/C E-TEK (black) catalysts at the cathode under the operating conditions described.

Figure 6 summarizes the cell voltage corrected by the ohmic drop (iR-free) at 0.5 A·cm^−2^ for the three most performing catalysts at the different operating conditions used. Although a comparison with the literature is not easy due to the different conditions and materials (also in terms of catalyst loading) used, we can observe that the performance is similar to that recorded in Refs. [8,30,31], where potential values (iR-free) around 0.8 V are reported at 0.5 A·cm^−2^. Mani et al. [8] reported a cell voltage lower than 0.75 V for a PtNi/C cathode-based MEA at 80 °C, 100% RH, H_2_/O_2_ and Pt loading close to 0.1 mg·cm^−2^ at a much lower current density of 0.002 A·cm^−2^. Da Silva et al. [31] employed a higher Pt loading of 0.5 mg·cm^−2^ for several PtNi catalysts. The best-performing one achieved a cell voltage of 0.34 V at 1000 A·g_Pt_^−1^, equivalent to 0.5 A·cm^−2^ (reported in Figure 6), but not iR-corrected, at 80 °C and fully humidified H_2_/O_2_ streams. The series resistance was quite high (0.94 Ω·cm^2^) and, after iR correction, the cell voltage is close to 0.81 V. Peng et al. [30] have recently reported PEFC results for a PtNi catalyst performing 0.8 V at 0.5 A·cm^−2^ with 75% RH at 80 °C. Han et al. [29] obtained very promising results using dealloyed Pt-Ni catalysts. The dealloying method is a useful approach to obtain Pt-rich surface or core-shell structures for bimetallic catalysts [32]. They showed high performance and durability using a family of dealloyed Pt-Ni catalysts for the ORR [29,32]. The behavior at 80 °C and low current density were comparable to that observed in the present work using Pt_3_Ni_2_/C and Pt_1_Ni_1_/C catalysts, although 0.2 mg·cm^−2^ Pt loading was used in our investigation compared to 0.1 mg·cm^−2^ reported in Ref. [29]. At high current densities, the performance reached by Han et al. [29] was higher compared to that obtained in the present analysis with our Pt-Ni catalysts; however, it must be taken into account that a thinner membrane was employed in Ref. [29], which produced a lower cell resistance.

Furthermore, in order to increase the stability of the catalysts, a post-dealloying thermal annealing is usually applied [29,32]. This procedure produces an increased Pt surface diffusion rate promoting an improved Pt-skin layer on the bimetallic catalyst [29,32]. Furthermore, the dealloying process, in particular starting from high initial non-noble atom concentration, generates porous structure, clearly visible by scanning tunneling electron microscopy (STEM) [29,32]. In our approach, the thermal treatment was carried out before the acid leaching procedure, and, in the case of the Pt_1_Ni_1_/C catalyst, produced a more ordered alloy structure, as observed from the XRD pattern. From TEM images reported in Figure 2 at low magnification, porous nanoparticles are not detectable, and, although a Pt enrichment of the surface can be envisaged from XPS, the formation of a Pt skin layer should be confirmed by using other techniques. In terms of performance, the formation of a more ordered crystallographic structure for the Pt_1_Ni_1_/C catalyst did not translate into a better behavior; however, this, together with the possible formation of a Pt skin layer, could be the reason of the improved stability obtained for the cell based on this catalyst (see discussion below). This was also reported in a recently published paper [33], in which the conversion of PtNi nanoparticles from a disordered solid solution to an ordered intermetallic compound led to an enhanced durability and better ORR activity. In our case, the ORR activity was found to be better for the Pt_3_Ni_2_ disordered solid solution compared to the ordered Pt_1_Ni_1_, more likely due to a major presence of Pt in the catalyst.

**Figure 7:**
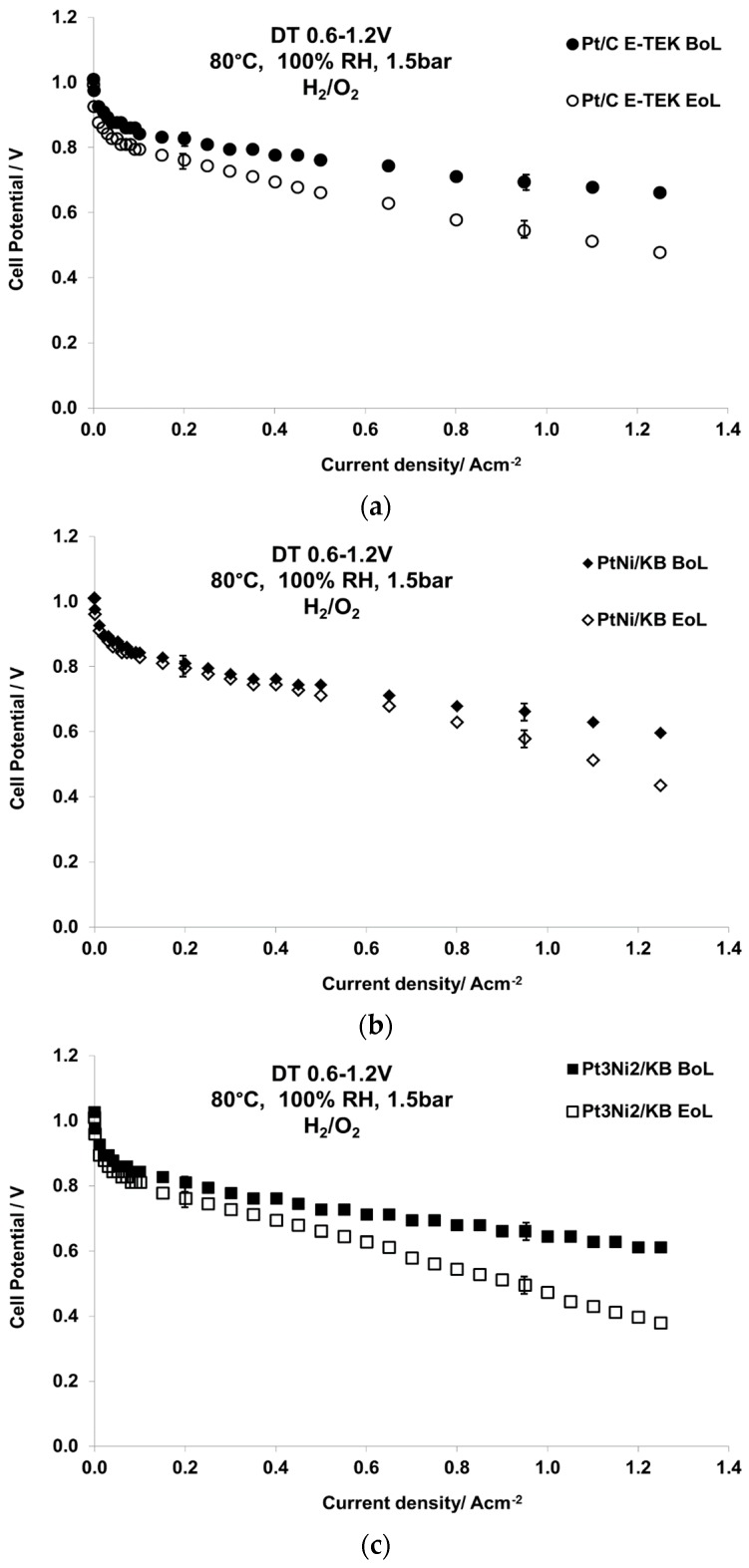
Polarization curves before and after the ADT for the MEAs based on carbon supported (**a**) Pt; (**b**) Pt_1_Ni_1_; and (**c**) Pt_3_Ni_2_ at 80 °C, 100% R.H., 1.5 bar_abs_, H_2_/O_2_.

Accelerated degradation tests (ADTs), i.e., 15,000 step cycles (steps 0.6–1.2 V, cycle time 6 s, H_2_–N_2_), were carried out for all catalysts at 80 °C, 100% R.H., 1.5 bar_abs_. A milder protocol, i.e., 0.6–0.9 V cycling, was first carried out, but the differences in performance before and after the test were found to be almost negligible, as observed in Figure S1 of the supporting information. After the degradation test, polarization curves were carried out under the same conditions feeding oxygen at the cathode (Figure 7). From the analysis of the curves recorded before and after the degradation test, it appears that the equimolar bimetallic catalyst (Pt_1_Ni_1_) shows the best stability since the polarization profile does not change much after the ADT, especially in the low current density region.

**Figure 8:**
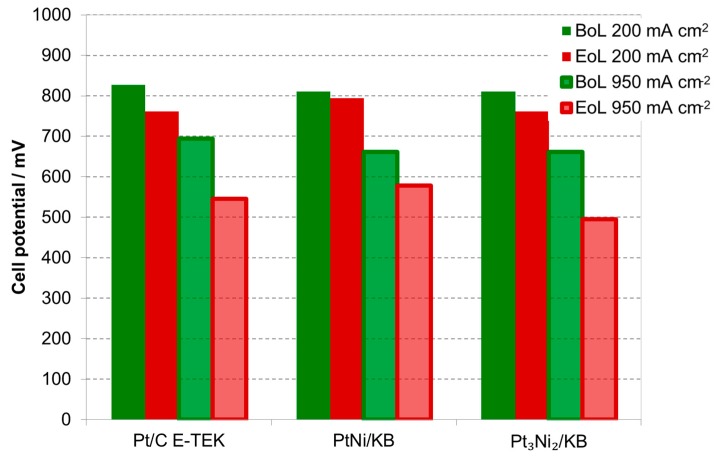
Histogram reporting the cell potential at 200 and 900 mA·cm^−2^ for the MEAs based on the different cathodic catalysts before and after the ADT.

The benchmark catalyst shows the largest losses in performance, as can be derived from the histograms reported in Figure 8, which summarizes the potential losses at two values of current density (of interest for what concerns catalytic activity and practical application) of the various MEAs after the ADT. Figure 8 shows the cell potential at 200 mA·cm^−2^ and 950 mA·cm^−2^ for the three MEAs subjected to the ADT. The MEA based on the benchmark Pt catalyst showed the largest potential losses at low current density (8%) after the ADT while the Pt_1_Ni_1_/C-based MEA was the most stable (2% and 12.5% voltage losses at 200 mA·cm^−2^ and 950 mA·cm^−2^, respectively). In all cases, the latter MEA presented the highest cell potential after the ADT among the investigated ones both at 200 and 950 mA·cm^−2^. Cyclic voltammetry analyses carried out before and after the ADT (not shown) allowed the determination of the electrochemical active surface area (ECSA) that is reported in Table 4, together with the crystallite size determined by XRD at the BoL and EoL. The values show that all catalysts are affected by significant particle sintering; the crystallite size increases from three to five times after the tests, causing a relevant decrease of ECSA to almost half the initial value. However, the catalyst showing a more ordered crystallographic structure appears less affected by sintering phenomena during the ADT [33]. It is worth mentioning that the ECSA is higher for the PtNi/C catalysts, related to their smaller particle size as revealed by XRD and TEM analyses. No significant change in the ECSA at the BoL may be attributed to the different content in Ni between PtNi/KB and Pt_3_Ni_2_/KB. Da Silva et al. reported an increased ECSA when introducing Ni caused by the decrease of Pt–Pt bond distance (geometric effect) and the increase of the 5d-band vacancy (electronic effect) [31].

**Figure 9:**
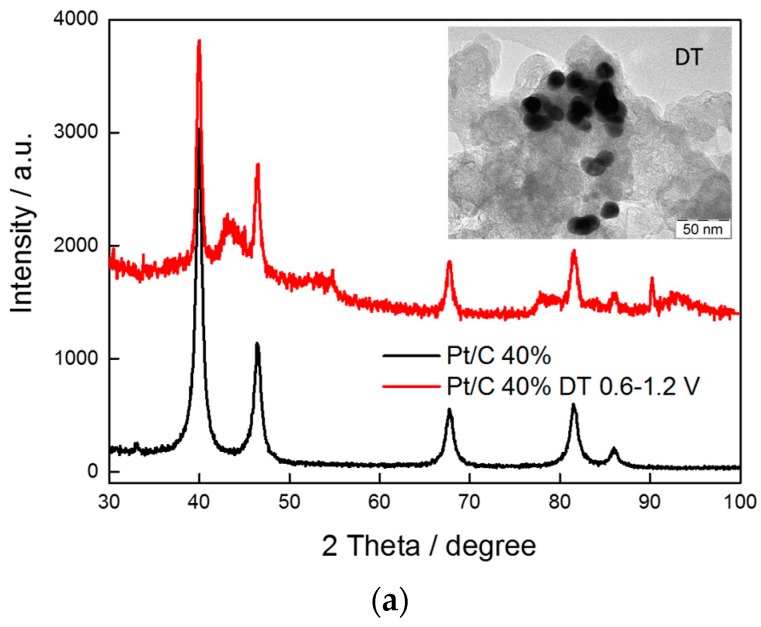

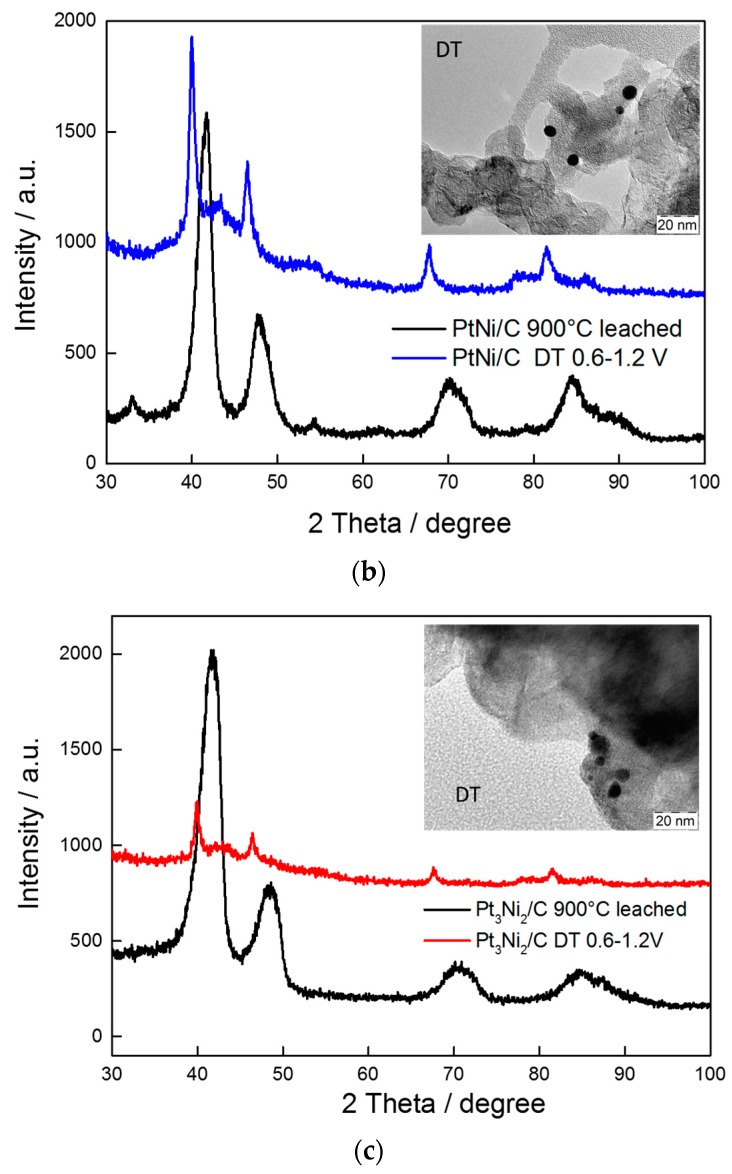
XRD patterns of carbon supported (**a**) Pt/C; (**b**) PtNi1C; (**c**) Pt_3_Ni_2_/C catalysts before and after the ADT. The inset shows the TEM image of the catalyst after the ADT.

To confirm this conjecture, XRD and TEM analyses were carried out after the ADTs; the XRD patterns and the TEM images after the ADTs are reported in Figure 9. A dramatic increase of crystallite size can be observed in the XRD patterns, together with a significant shift of the peaks towards lower Bragg angles, which is an indication of a dealloying phenomenon. From TEM micrographs, we can derive that a particle sintering occurred together with a leaching of particles due to carbon corrosion (as can be observed in Figure S2 of the supplementary information). Thus, it appears that this preparation procedure is not suitable to obtain stable catalysts. Recently, Pt-Ni nanocage (PNC) catalysts were synthesized by a solvothermal method and investigated in PEFCs in terms of performance and stability [30]. The PNC experienced slight agglomeration and some edge loss after cycling (30 K cycles in the range 0.6 V to 1.0 V). However, the majority of the particles retained their cage structure. In our case, large agglomeration and particles losses were observed indicating that the high temperature treatment is not enough to stabilize the PtNi alloy, in particular under these severe testing conditions (0.6–1.2 V cycling). Under milder conditions (0.6–0.9 V cycling) the results envisaged lower stability constraints (see Figure S1 in the supplementary information).

## 3. Materials and Methods

### 3.1. Catalyst Preparation and Physico-Chemical Characterization

Carbon supported Pt-Ni catalysts with different atomic Pt:Ni ratios (1:1, 3:2 and 2:3) were prepared by the formic acid reduction [34] method using a 0.5 M HCOOH concentration. Ketjenblack (KB) from AzkoNobel was used as the carbon support. A suspension of KB in 0.5 M formic acid solution was heated at 80 °C. Chloroplatinic acid (H_2_PtCl_6_. 6H_2_O) and Nickel nitrate (Ni(NO_3_)_2_. 6H_2_O) solutions were slowly added to the carbon suspension. The slurry was maintained at 80 °C for 5 h. The suspension was left to cool at room temperature and the solid filtered and dried in an oven at 100 °C for 2 h. The total nominal metal concentration was 50 wt %. The as-prepared catalysts were thermally treated at 900 °C in inert (Ar) atmosphere and a related structural analysis was carried out. A pre-leaching procedure at 80 °C in 0.5 M HClO_4_ was carried out for the PtNi catalysts after the thermal reduction. Physical properties such as the lattice parameter and the average crystallite size were determined by using a powder X-ray diffraction (XRD) technique. XRD patterns were obtained using a Philips X’Pert X-ray diffractometer equipped with a Cu/Kα source operating at 40 kV and 30 mA. Scans were carried out at 2° min^−1^ for 2θ values between 10° and 100°.

The catalyst composition of the different powder samples was determined by energy dispersive X-ray analysis (EDX) using an FEI XL 30-Feg SEM-EDX instrument (FEI, Eindhoven, The Netherlands). Transmission electron micrographs (TEM) of the PtNi catalysts were obtained using a Philips CM12 transmission electron microscope (Philips, Eindhoven, The Netherlands) with spatial resolution of 0.2 nm. For this analysis, the catalysts were ultrasonically dispersed in isopropyl alcohol and deposited onto copper grids covered with carbon films. To obtain the electron micrographs, the catalyst samples were finely grinded and an amount of 200 metal particles was measured to obtain a particle size distribution histogram for each catalyst.

X-ray photoelectron spectroscopy (XPS) measurements was carried out using a Physical Electronics (PHI) 5800-01 spectrometer (Chanhassen, MN, USA). A monochromatic Al Kα X-ray source was used at a power of 350 W. Spectra were obtained with pass energies of 58.7 eV for elemental analysis (composition) and 11.75 eV for the determination of the oxidation states. The pressure in the analysis chamber of the spectrometer was 1 × 10^−9^ Torr during the measurements. The Ag 3d_5/2_ peak of an Ag foil was taken, after argon sputtering, for checking the calibration of the binding energy (BE) scale. The quantitative evaluation of each peak was obtained by dividing the integrated peak area by atomic sensitivity factors, which were calculated from the ionization cross-sections, the mean free electron escape depth and the measured transmission functions of the spectrometer. XPS data have been interpreted by using the on-line library of oxidation states implemented in the PHI MULTIPAK 6.1 software (version 6.1, Physical Electronics, Chanhassen, MN, USA) and the PHI Handbook of X-ray photoelectron spectroscopy [35].

### 3.2. Electrodes and MEA Preparation

To evaluate the performance of the electro-catalysts, different electrodes were prepared by using as cathode electro-catalyst the Pt–Ni alloys previously prepared. For the sake of comparison, a commercial 40% Pt/C E-TEK was selected to investigate the performance and durability in order to have similar electrode features (i.e., thickness, Pt:C ratio, etc.). At the anode side, an in-house prepared Pt/C catalyst was used in all experiments [36]. The catalytic ink was prepared by mixing, in an ultrasonic bath, the electro-catalyst with a 33 wt % of dry Nafion (5 wt % hydroalcoholic solution) as a ionomer [37]. For catalyst layer preparation a 20 wt % of ammonium carbonate (Carlo Erba, Milan, Italy) was used as a pore-former. The catalytic ink was deposited onto a gas diffusion layer SGL25BC, by a standardised spray technique [38]. A Pt loading of 0.2 mg/cm^2^ was used for both electrodes. The MEAs were prepared by hot pressing, assembling the electrodes with a commercial NR212 membrane at 125 °C. 

### 3.3. Electrochemical Studies

Electrochemical studies were performed in a 25 cm^2^ single cell, in a temperature range between 80 °C and 95 °C, at a pressure ranging from 3 to 1.5 bar_abs_ and at different relative humidities (R.H.), from 100% to 50%. The cell was fed with H_2_ as fuel and air or O_2_ as oxidant in order to evaluate simultaneously the single cell performance and the catalytic activity for the ORR. The flow rates were varied to have constant stoichiometry of 2 and 1.5 for oxidant and fuel, respectively. The single cell performance was investigated by steady-state galvanostatic measurements. The cell was connected to a fuel cell test station including an HP6051A electronic load. For the cyclic voltammetry (CV) studies, the single cell was connected to a Potentiostat/Galvanostat PGSTAT30 AUTOLAB Metrohm (Utrecht, The Netherlands), equipped with a frequency response analyzer (FRA) module and a 20 A current booster. Humidified hydrogen was fed to the anode side, which operated as both counter and reference electrode, whereas humidified nitrogen was fed to the cathode side (working electrode) at 1 bar_abs_. The sweep rate was 50 mVs^−1^. The electrochemical active surface area was determined by integration of the CV profile in the hydrogen adsorption region after correction for the double layer capacitance. Data were not corrected for ohmic drop and hydrogen cross-over. The cell resistance was measured at open circuit voltage by using a Hewlett-Packard (type HP 4338B) milli-ohmmeter (Hewlett-Packard, Palo Alto, CA, USA), working at a frequency of 1 kHz. Moreover, in order to verify the electro-catalysts stability, accelerated degradation tests, at 80 °C, 100% R.H., 1 bar_abs_ H_2_–N_2_, were carried out by cycling the cell potential between 0.6 V and 1.2 V. The performance analyses were carried out by comparing electrochemical and physico-chemical parameters at the beginning of life (BoL) and end of life (EoL).

## 4. Conclusions

PtNi electrocatalysts were prepared by the formic acid method and successive thermal and leaching treatments in order to assess the best composition in terms of catalytic activity, fuel cell performance and stability. The catalyst characterized by a larger Pt content (Pt_3_Ni_2_/C) presented the highest catalytic activity during the polarization curves (lower potential losses in the activation region) among the synthesized and reference commercial catalysts at high temperature and low humidification conditions typical of automotive applications. Accelerated degradation tests carried out, by using a severe cycling protocol, on the MEAs equipped with the bimetallic in-house PtNi catalysts and the benchmark Pt/C demonstrated that the equimolar PtNi catalyst was the most stable formulation showing the best performance at the end of life (0.58 V at 950 mA·cm^−2^). Thus, it appeared that both the crystalline features (i.e., ordered crystallographic phase and 2.7 nm as size) and a larger presence of Ni contributed to the enhanced performance of the 50% PtNi/C after the ADT, although a significant loss of activity and ECSA were recorded due to carbon corrosion, particle sintering and Ni dealloying.

## Figures and Tables

**Table 1:** Physico-chemical properties of prepared catalysts.

Sample Catalyst	Position (200), ° 2θ	Crystallite Size, nm	Lattice Parameter (200), nm	X_Ni_ Alloy, %
PtNi/KB	70.348	2.7	0.378	35
Pt_3_Ni_2_/KB	70.608	2.3	0.377	38
Pt_2_Ni_3_/KB	69.617	6.5	0.382	26

**Table 2:** Physico-chemical properties of prepared catalysts.

Sample	Chemical States	Relative Peak Area (%)	BE (eV)
**PtNi/KB**			4f_7/2_
	Metallic Pt	48.19	71.11
	Pt^2+^	51.81	71.49
			2p_3/2_
	Metallic Ni	2.53	853.38
	NiO	3.29	855.18
	NiOOH	70.95	856.28
	Ni(OH)_2_	23.23	857.71
**Pt_3_Ni_2_/KB**			4f_7/2_
	Metallic Pt	16.88	71.51
	Pt^2+^	70.88	71.80
	Pt^4+^	12.24	73.46
			2p_3/2_
	Metallic Ni	19.74	852.86
	NiO	7.19	854.74
	NiOOH	36.64	856.12
	Ni(OH)_2_	36.43	862.16
**Pt_2_Ni_3_/KB**			4f_7/2_
	Metallic Pt	3.96	71.34
	Pt^2+^	88.38	71.43
	Pt^4+^	7.66	73.27
			2p_3/2_
	Metallic Ni	5.67	854.18
	NiO	34.84	855.75
	NiOOH	23.35	857.06
	Ni(OH)_2_	36.14	858.71

**Table 3:** Summary of the fuel cell results.

Operating Conditions	Sample Catalyst	OCV, V	R Cell, Ω·cm^2^	E @ 500 mA·cm^−2^, V
**80 °C, 100% RH, 3 bar_abs_, H_2_/air**	Pt/C E-TEK	0.976	0.067	0.744
	PtNi/KB	0.982	0.127	0.717
	Pt_3_Ni_2_/KB	0.993	0.107	0.744
	Pt_2_Ni_3_/KB	0.943	0.250	-
**80 °C, 100% RH, 3 bar_abs_, H_2_/O_2_**	Pt/C E-TEK	0.993	0.070	0.794
	PtNi/KB	1.015	0.118	0.767
	Pt_3_Ni_2_/KB	1.009	0.097	0.777
	Pt_2_Ni_3_/KB	1.009	0.199	0.495
**80 °C, 50% RH, 1.5 bar_abs_, H_2_/O_2_**	Pt/C E-TEK	0.993	0.082	0.761
	PtNi/KB	0.999	0.139	0.733
	Pt_3_Ni_2_/KB	1.026	0.103	0.761
**95 °C, 50% RH, 2 bar_abs_, H_2_/O_2_**	Pt/C E-TEK	0.960	0.075	0.744
	PtNi/KB	1.000	0.112	0.733
	Pt_3_Ni_2_/KB	1.000	0.084	0.777

**Table 4:** Electrochemical active surface area and crystallite size at the beginning and end of life for the different MEAs.

Sample Catalyst	Status	ECSA, m^2^·g^−1^	Crystallite Size, nm
**Pt/C E-TEK**	BoL	22	5
	EoL	9	16
**PtNi/KB**	BoL	44	2.7
	EoL	18	12
**Pt_3_Ni_2_/KB**	BoL	42	2.3
	EoL	12	15

## Supplementary Materials

The following are available online at www.mdpi.com/1996-1944/10/3/317/s1.
