# Supplementary material for: Towards Highly Performing and Stable PtNi Catalysts in Polymer Electrolyte Fuel Cells for Automotive Application
Source: Materials (Basel). 2017 Mar 21;10(3):317. doi: 10.3390/ma10030317 (PMC5503373; doi:10.3390/ma10030317)
Supplement: Supplementary file 1 [file materials-10-00317-s001.pdf]

Supplementary information

Towards Highly Performing and Stable PtNi Catalysts in Polymer Electrolyte Fuel Cells for Automotive Application

Sabrina C. Zignani, Vincenzo Baglio *, David Sebastián, Ada Saccà, Irene Gatto and Antonino S. Aricò

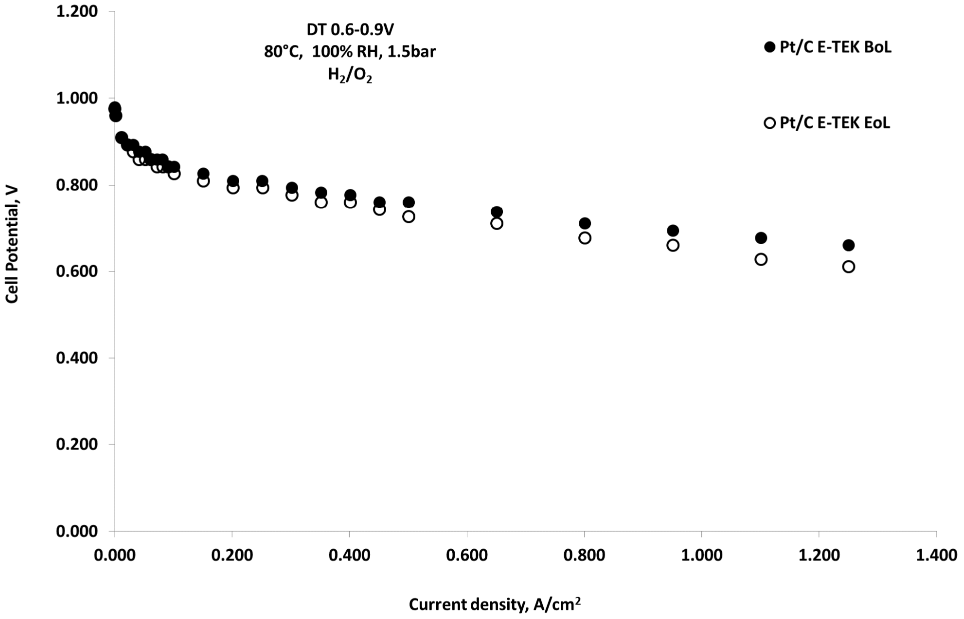

(a)

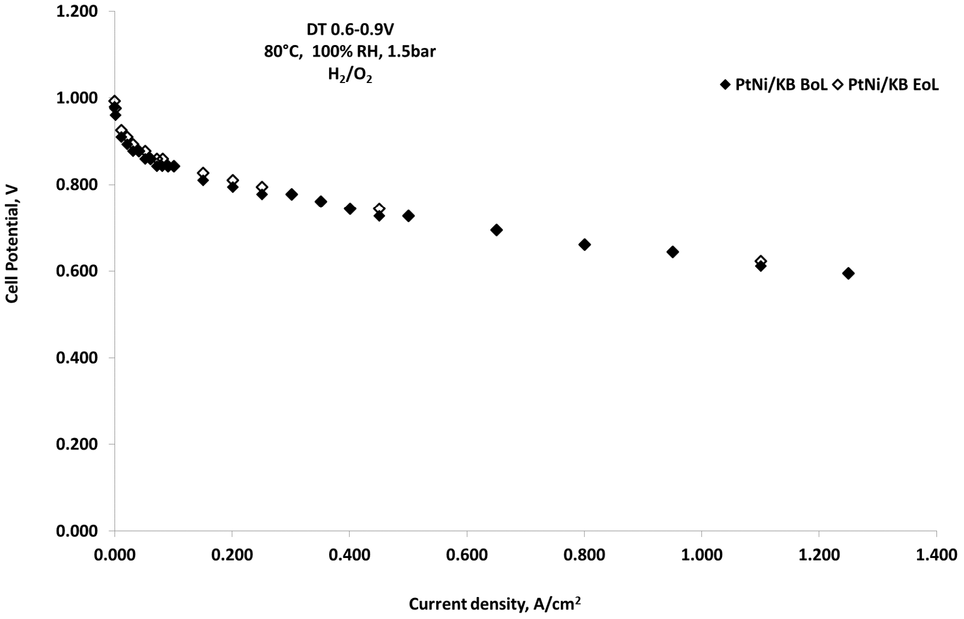

(b)

Figure S1. Polarization curves before and after the mild ADT (0.6–0.9 V cycling) for the MEAs based on carbon supported (**a**) Pt; and (**b**) Pt1Ni1, at 80°C, 100% R.H., 1.5 barabs., H_2/O_2.

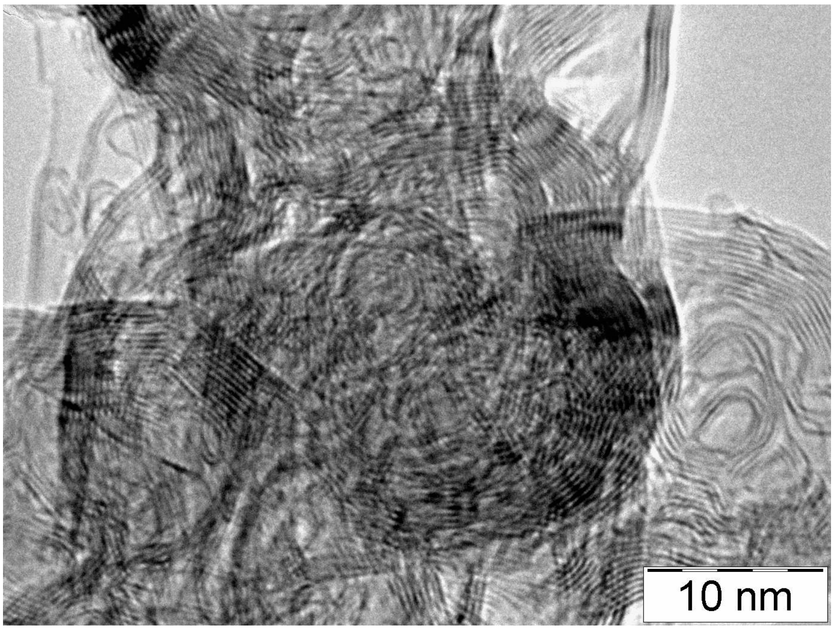

Figure S2. High-magnification TEM image of the PtNi/C catalyst after the severe ADT (0.6–1.2 V cycling).
